# Association between physical activity and sedentary behavior and gestational diabetes mellitus: a Mendelian randomization analysis

**DOI:** 10.3389/fendo.2024.1389453

**Published:** 2024-12-16

**Authors:** Jie Gao, Jingfang Liu

**Affiliations:** ^1^ The First Clinical Medical College, Lanzhou University, Lanzhou, Gansu, China; ^2^ Department of Endocrinology, the First Hospital of Lanzhou University, Lanzhou, Gansu, China

**Keywords:** gestational diabetes mellitus, mediation mendelian randomization, multivariate Mendelian randomization, television watching, body mass index

## Abstract

**Introduction:**

The evidence of association between physical activity (PA), sedentary behavior (SB) and gestational diabetes mellitus (GDM) remains controversial in observational studies, this study aimed to generate new hypotheses between PA, SB and GDM.

**Methods:**

Our study performed Mendelian randomization (MR) analysis to explore the effects of three types of PA (moderate physical activity (MPA), moderate to vigorous physical activity (MVPA), accelerometer-based physical activity (ABPA)), three types of SB (television watching (TV), leisure computer use (PC), driving (DR)) on GDM and the mediating effect of body mass index (BMI). The inverse variance weighted method was used for the major analysis.

**Results:**

In univariate MR analysis, we found that genetically predicted TV and PC among SB were associated with GDM (OR = 1.61, 95%CI 1.21-2.14, *P* = 0.001; OR = 0.71, 95%CI 0.51-0.98*, P* = 0.037), whereas DR and MP were not (OR = 1.68, 95%CI 0.21-13.3, *P* = 0.623; OR = 1.20, 95%CI 0.87-1.65, *P* = 0.271). However, no association was found between physical activity and GDM: MPA (OR = 0.40, 95%CI 0.08-2.06, *P* = 0.273), MVPA (OR = 0.96, 95%CI 0.58-1.57, *P* = 0.861), and ABPA (OR = 0.99, 95%CI 0.90-1.09, *P* = 0.838). Multivariate MR analysis found DM (OR = 1.64, 95%CI 1.13-2.36, *P* = 0.008) and that BMI was a mediating factor with a 62% mediating effect.

**Conclusions:**

This study proposes a new hypothesis for the association between TV and GDM, which is mediated by BMI, providing evidence for reducing the risk of GDM during pregnancy by reducing television watching time.

## Introduction

Gestational diabetes mellitus (GDM) is a common metabolic disease of pregnancy that increases the risk of adverse pregnancy and neonatal outcomes (1). Several studies have thoroughly analyzed the risk factors for GDM, which include family history of diabetes, previous history of GDM, advancing maternal age, and obesity (2). In particular, obesity is recognized as one of the strongest risk factors for GDM, with higher body mass index (BMI) significantly increasing the likelihood of developing GDM. Women with a BMI of 25.0-29.9 kg/m² are classified as overweight, while those with a BMI ≥30 kg/m² are classified as obese, both categories being associated with elevated GDM risk (3, 4). Additionally, modern sedentary lifestyles, characterized by prolonged periods of physical inactivity and sedentary behavior (SB), are increasingly contributing to the rising incidence of GDM (5).

Physical inactivity, poor maternal sleep, and adverse psychological status have also been identified as contributing factors (6). Physical activity (PA) and sedentary behavior (SB), two important elements of intensive lifestyle interventions (ILI), are commonly cited in studies addressing the prevention of GDM (7, 8). PA increases glucose uptake, improves insulin sensitivity, and reduces the incidence of GDM (9, 10), particularly at moderate intensity (11). In recognition of these benefits, the American College of Obstetrics and Gynecology recommends that pregnant women engage in at least 150 minutes of moderate-intensity exercise per week (12). However, despite these guidelines, more than half of pregnant women fail to meet the recommended PA levels, and many spend the majority of their waking hours engaged in sedentary activities (13, 14). Sedentary behavior, particularly television watching (TV), has been independently linked to elevated blood glucose levels and an increased risk of maternal hyperglycemia, which are risk factors for GDM (15). Reducing SB has been associated with improved glycemic control, suggesting that limiting sedentary time could lower GDM risk (16).

Although increasing PA and reducing SB could theoretically influence weight management and reduce GDM risk, the relationship between PA, SB, and GDM remains controversial. Some large studies, such as RADIEL and DALI, have reported mixed findings on the efficacy of PA in reducing GDM incidence, and whether SB during pregnancy definitively increases GDM risk is still under debate (17–19). Some evidence suggests that PA during pregnancy may not significantly reduce GDM incidence (20), while others argue that SB is not consistently associated with GDM risk (21, 22). This discrepancy may be attributed to variations in the gestational periods and intervention times across studies, as well as confounding factors like diet and health perceptions.

Given these challenges, traditional observational studies are limited in their ability to infer associations between PA, SB, and GDM due to the influence of confounding biases and potential reverse causality. To address these limitations, Mendelian randomization (MR) has emerged as a robust genetic epidemiological approach. By using single-nucleotide polymorphisms (SNPs) as instrumental variables, MR allows for the testing of effect size between exposures (e.g., PA and SB) and outcomes (e.g., GDM), effectively mitigating the impact of confounding factors (23). Moreover, no prior MR studies between PA, SB, and GDM, leaving a significant gap in the literature.

Therefore, this is an explorative study aiming to generate new hypotheses for the possible association between PA, SB, and GDM using MR analysis. Specifically, we hypothesized that PA and SB are correlated with GDM, and that BMI may play a mediating role. Through MR analysis, we sought to determine the proportion of the mediating effect of BMI between PA, SB, and GDM, providing a clearer understanding of the underlying mechanisms involved.

## Methods and materials

### Study design

**Figure 1:**
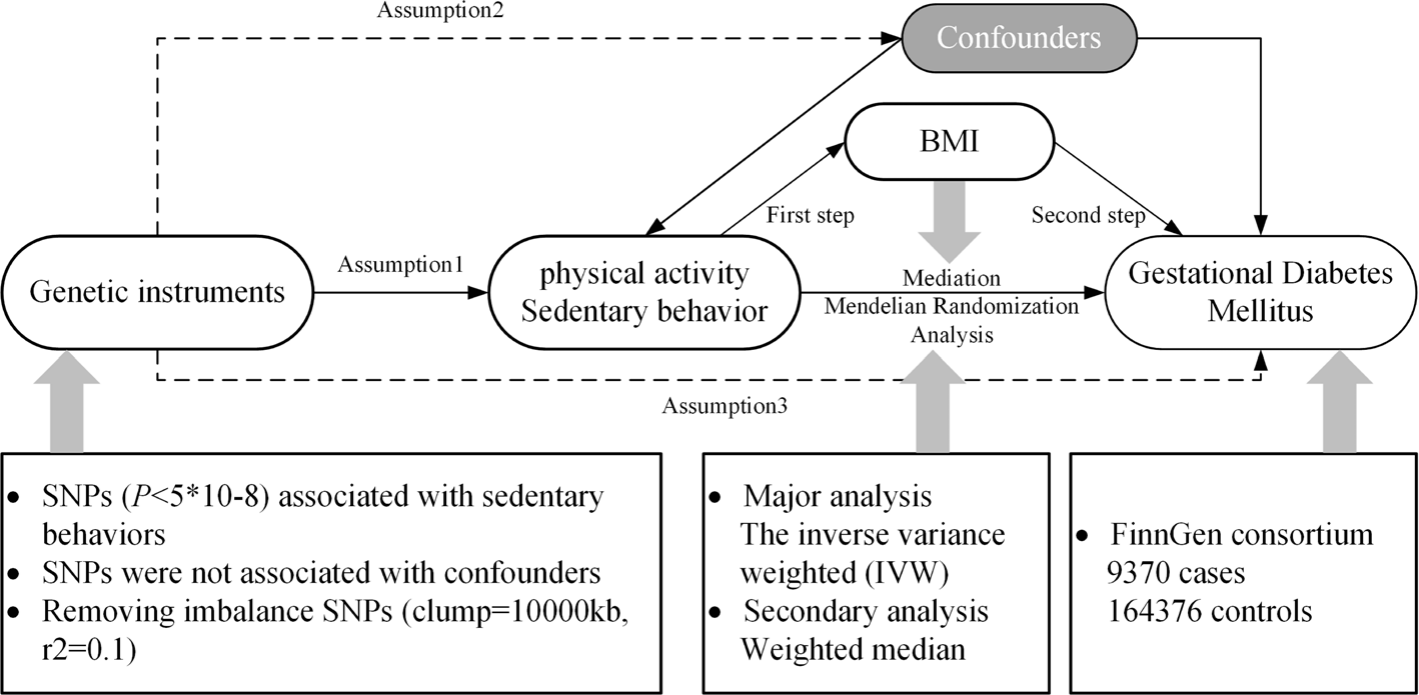
Research rationale and flow chart. BMI, body mass index; SNP, single-nucleotide polymorphism.

The principles of this study are shown in **Figure 1**, and a specific discussion of the underlying assumptions of MR has been provided in a previous study (24). We selected three types of PA: moderate PA (MPA), moderate-to-vigorous PA (MVPA), and accelerometer-based PA (ABPA) and three types of SB: TV, leisure computer use (PC), driving (DR) and mobile phones use (MP). These six different traits were regarded as exposures and GDM as the outcome, and a univariate MR (UVMR) analysis was performed. After completing these analyses, a multivariate MR (MVMR) was performed after integrating the exposures that were significantly associated with the outcome, GDM. Subsequently, we used the “two-step” method to evaluate whether the exposure affects GDM through the mediation of BMI. BMI was measured at the first prenatal visit, and weight gain during pregnancy was not directly measured, but was inferred through its association with behaviors such as television watching.

### Data source

#### Exposure and mediator

The genome-wide association study (GWAS) summary statistical data of all exposures used in this study were retrieved from the largest research (25, 26). PA is mainly defined by questionnaires: (1) For MPA, participants were asked: “In a typical WEEK, on how many days did you do 10 minutes or more of moderate physical activities like carrying light loads, cycling at normal pace? (Do not include walking)”. We assessed PA during the first trimester of pregnancy, as early pregnancy provides a critical baseline for evaluating lifestyle factors that may influence gestational outcomes. PA levels tend to decline as pregnancy progresses due to increasing physical discomfort and other factors. Evaluating PA in early pregnancy allows us to capture habits that may have a significant impact on the development of GDM. The implications of assessing PA at different stages of pregnancy are crucial: while PA in early pregnancy may reflect pre-pregnancy behavior, PA in the second and third trimesters is often more limited, which can diminish its potential protective effects against GDM. (2) For MVPA, this activity was calculated by taking the sum of total minutes/week of MPA multiplied by four and the total number of vigorous PA (which also defined by questionnaires) minutes/week multiplied by eight, corresponding to their metabolic equivalents. (3) For ABPA, participants were told to wear an Axivity AX3 wrist-worn accelerometer and began monitoring for up to 7 days, physical activity information was extracted from 100Hz raw triaxial acceleration data after calibration, removal of gravity, sensor noise, and identification of wear/non-wear episodes. Individuals with less than 72 hours of data or no data for each hour of a 24-hour period and outliers with values more than 4 standard deviations above the mean were excluded. SB was also determined by asking three questions: (1) For TV, participants were asked: “In a typical DAY, how many hours do you spend watching TV?”; (2) For PC, participants were asked: “In a typical DAY, how many hours do you spend using the computer? (Do not include using a computer at work)”. (3) For DR, participants were asked: “In a typical DAY, how many hours do you spend driving?”. The duration of these sedentary behaviors was treated as exposure measurements. (4) For MP, participants were asked: “In a typical DAY, how many hours do you spend using mobile phones?”. The duration of these sedentary behaviors was treated as exposure measurements. The GWAS summary statistical data for BMI were obtained from the IEU Open GWAS Project (https://gwas.mrcieu.ac.uk/), and the corresponding GWAS ID was “ukb-b-19953.” The specific data sources are listed in **Table 1**.

**Table 1:** Overview of GWAS data used in MR.

Traits	Year	Author	Consortium	Unit (SD)	Sample size	Anccestry	PMID
MPA	2018	Klimentidis et al	UKBB	NA	343,827	European	29899525
MVPA	2018	Klimentidis et al	UKBB	2,084 (MET)	377,234	European	29899525
ABPA	2017	Doherty et al	UKBB	8.14 (milli-gravities)	91,084	European	28146576
TV	2020	van de Vegte et al	UKBB	1.5 (h)	437,887	European	32317632
PC	2020	van de Vegte et al	UKBB	1.2 (h)	360,895	European	32317632
DR	2020	van de Vegte et al	UKBB	1.0 (h)	310,555	European	32317632
MP	2020	Ben Elsworth	MRC-IEU	NA	456,972	European	–
BMI	2018	Ben Elsworth	MRC-IEU	4.8 (kg/m^2^)	461,460	European	–

MPA, moderate physical activity; MVPA, moderate to vigorous physical activity; ABPA, accelerometer-based physical activity; TV, television watching; PC, leisure computer use; DR, driving; MP, mobile phone use. SD, standard deviation; MET, metabolic equivalents.

#### Outcome

GWAS summary statistical data for GDM were obtained from the latest R7 release of the FinnGen GWAS results (https://r7.finngen.fi/). The corresponding phenotypic codes obtained were “GEST_DIABETES”, including 173,746 individuals (9,370 cases and 16,437 controls) of European ancestry. The GDM population was defined using Finnish health and population registry sources including registry data from inpatient hospitalizations, outpatient specialty clinics, and birth registry with diagnosed with ICD9, ICD10 within the gestational window (40 weeks before to 5 weeks after delivery) and excluding diabetes diagnosed before the first pregnancy (27).

### UVMR

The R package “TwoSampleMR” (version 0.5.6) in the R software (version 4.1.3) was used for analysis. For instrumental variable selection, the SNPs selection criteria were as follows: genome significance threshold, *P* < 5×10^8^; linkage disequilibrium threshold, r^2^ < 0.1; and clump = 10,000 kb. The R^2^ values and F-statistics of the screened SNPs were calculated as follows: R^2  ^= 2 × EAF × (1 − EAF) × β^2^ (EAF, the effect allele frequency for each SNP; β, β coefficient for effect size on exposure) (28), F = R^2^ × (N − 2)/(1 − R^2^) (N, the sample size) (29). F-statistic >10 indicates a strong association between SNPs and exposure. In statistical analyses, we used the inverse variance weighted (IVW) method to assess the effect size between exposure and outcome, which provided the highest estimated power and chose the weighted median method for supplementary analysis (30). Abnormal outlier SNP were screened and excluded using the MR-pleiotropy residual sum and outlier test (MR-PRESSO) (31). The Cochrane’s Q test was used to evaluate the heterogeneity of the selected SNPs, and *P* < 0.05 indicated the existence of heterogeneity. Horizontal pleiotropy was assessed using the MR-Egger intercept, with *P* < 0.05 indicating the presence of pleiotropy and the deviation of the intercept from zero indicating the direction of pleiotropy. Leave-one-out analysis was performed to determine whether the stability of the results was affected by a single SNP. All *p* values were two-sided.

### MVMR

For instrumental variable integration, we used the SNPs union of exposures that had a significant association with the outcome of UVMR and other exposures in the same group. In the statistical analyses, we first adjusted for the effect of the integration of exposure and then used the least absolute shrinkage and selection operator (LASSO) regression to eliminate collinearity among multiple exposures. Additionally, the IVW method was used to evaluate the independent effect of a single exposure on GDM after adjustment. Statistical significance was defined as *p* < 0.05, and all *p* values were two-sided.

### Mediation MR analysis

In the first step, we used UVMR to calculate the effect (β1) of the screened exposure on BMI and that of BMI on the outcome; in the second step, we used MVMR to calculate the effect of BMI on GDM after adjusting for the screened exposures (β2) and calculated the effect of the screened exposures on GDM after adjusting for BMI (β3). If BMI had a mediating effect between the exposure and outcome, the proportion of the effect mediated by BMI was estimated using the following equation (32):


$$E\;\left(\%\right)=\frac{\sum_{K=1}^{K}\beta 1*\beta 2_{k}}{\sum_{K=1}^{K}\beta 3+\beta 1*\beta 2_{k}}$$

where, K refers to the mediator k (k=1 in this study, which was BMI). All β value were derived from MR instrumental analysis using the IVW method.

## Results

### UVMR analysis uncovered associated factors for GDM

**Figure 2:**
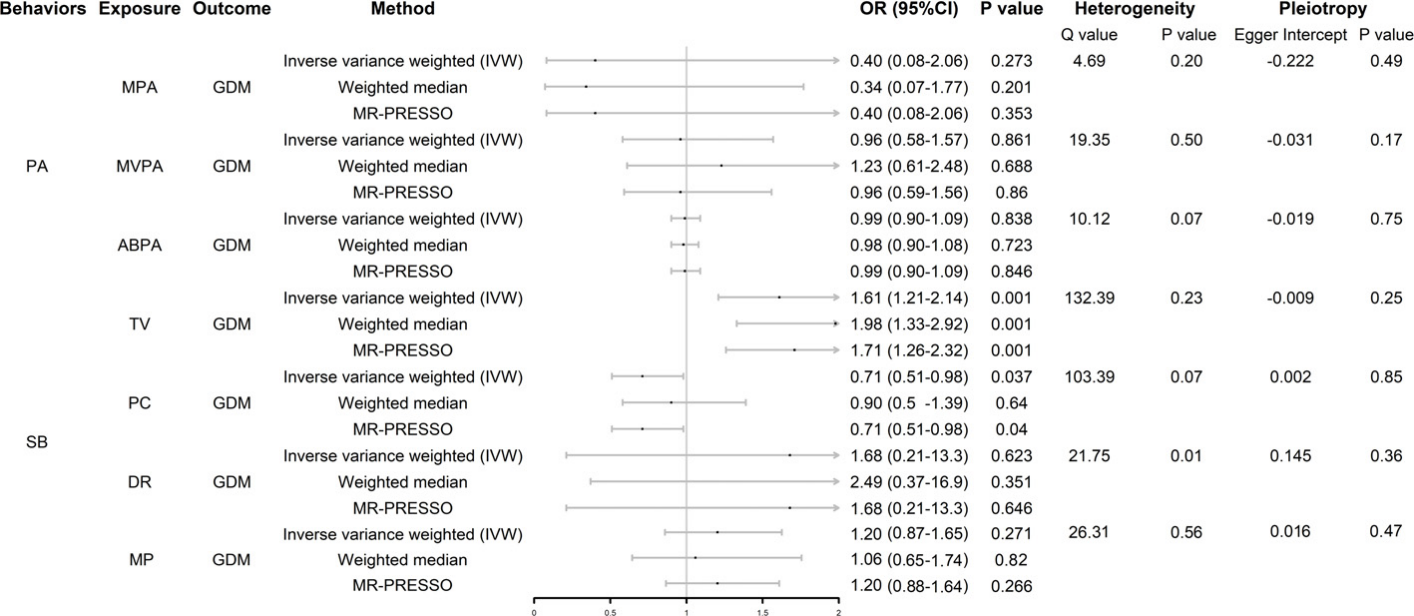
Forest plot of UVMR analysis. OR (95%CI) is the odds ratio of each MR estimate. P value is the significant level of each MR estimate. PA, physical activity; SB, sedentary behavior; GDM, gestational diabetes mellitus; MPA, moderate physical activity; MVPA, moderate to vigorous physical activity; ABPA, accelerometer-based physical activity; TV, television watching; PC, leisure computer use; DR, driving; MP, mobile phone use; IVW, inverse variance weighted method.

We screened SNPs closely related to exposure, including MPA4, MVPA22, ABPA6, TV123, PC85, MP31, and DR6, as instrumental variables. Details of the SNPs associated with each exposure included in the study are listed in **Supplementary Table S1**. As shown in **Figure 2**, there was no evidence of a hypothesized association between MPA, MVPA, and ABPA and GDM. In SB, genetically predicted TV and PC were associated with GDM (whereas DR and MP were not); however, the results of the weighted median model did not support the results of PC. The scatter plots are shown in **Supplementary Figure S1**.

In the heterogeneity analysis, the Cochran Q test results showed that there was no significant heterogeneity in the other exposure-outcome pairs, except DR-GDM, after correction using the MR-PRESSO test; however, the leave-one-out analysis showed that the result between this pair was not driven by a single SNP (**Supplementary Figure S2**). None of the exposure-outcome pairs had a significant pleiotropic effect (*p* > 0.05).

### MVMR analysis uncovered independent associated factors for GDM

**Figure 3:**
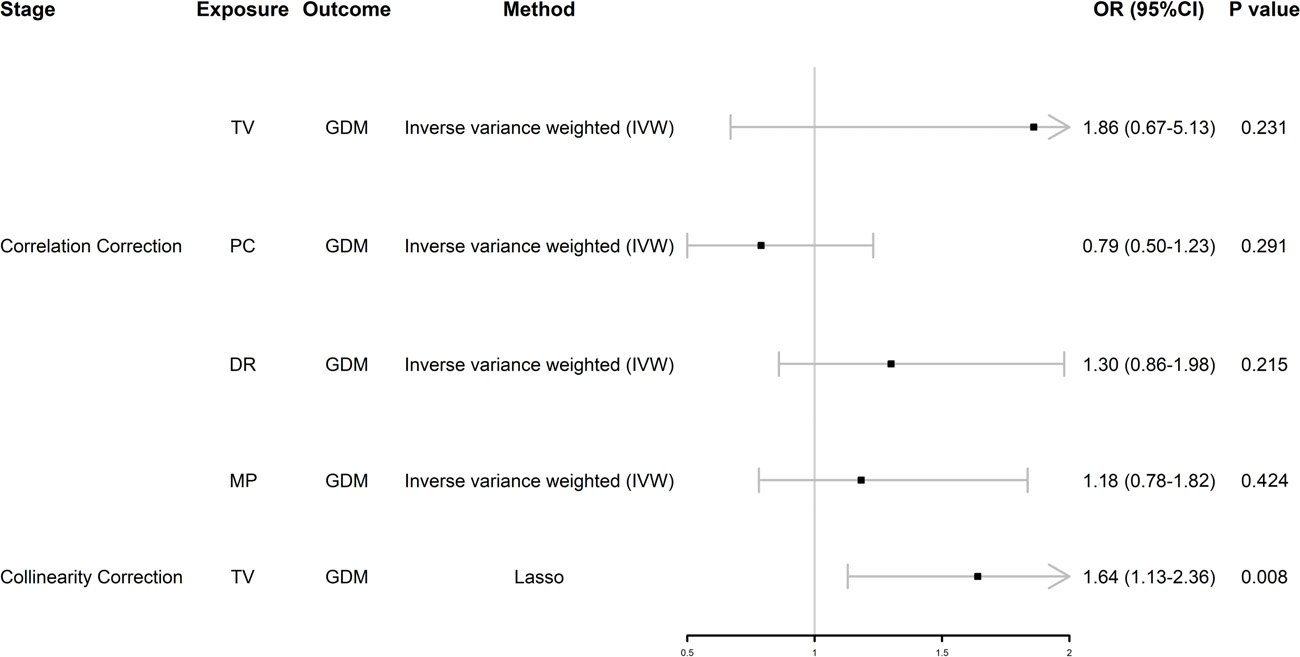
Forest plot of MVMR analysis. OR (95%CI) is the odds ratio of each MR estimate. P value is the significant level of each MR estimate. GDM, gestational diabetes mellitus; TV, television watching; PC, leisure computer use; DR, driving; MP, mobile phone use; Lasso, least absolute shrinkage and selection operator.

We found that TV and PC were significantly associated with GDM, so we performed MVMR analysis of SB. After initial correction for their interaction, we found that these four behaviors were not associated with GDM (**Figure 3**); however, after further removing collinearity, TV was positively associated with GDM independently of the other two types of SB, which means TV was an independent risk factor for GDM (odds ratio [OR] = 1.64, 95% confidence interval [CI] 1.13–2.36, *P* = 0.008) (**Figure 3**).

### Mediation MR analysis

**Figure 4:**
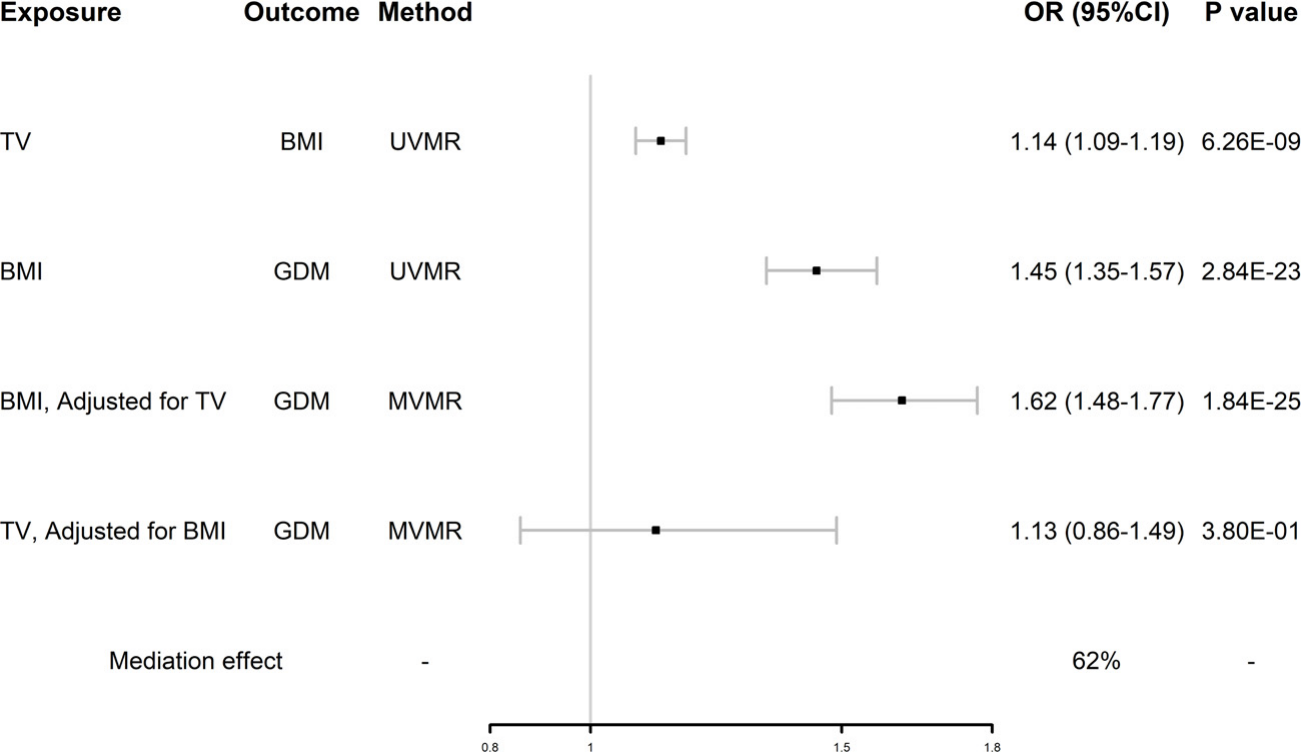
Forest plot of mediation MR analysis. GDM, gestational diabetes mellitus; TV, television watching; BMI, body mass index; UVMR, univariate Mendelian randomization; MVMR, multivariate Mendelian randomization.

After UVMR and MVMR analyses, we confirmed that TV had an independent hypothesized association with GDM and demonstrated whether BMI was a potential mediator between TV and GDM. As shown in **Figure 4**, in the first step, genetically predicted TV was positively associated with BMI, which was likewise positively associated with GDM. In the second step, after adjusting for TV, the direct effect of BMI on GDM remained positive. However, the direct effect of TV on GDM changed after adjusting for BMI. BMI mediated 62% of the effect of TV on GDM. By using this mediator index, we extrapolated the changes in BMI during pregnancy and detailed them in **Table 2**.

**Table 2:** Changes in BMI during pregnancy.

Time Point	BMI Measurement Timing	Mean BMI (kg/m²) ± SD	Normal BMI Range (18.5–24.9 kg/m²)	Overweight (25.0–29.9 kg/m²)	Obesity (≥30 kg/m²)
Pre-pregnancy	Before conception	24.9 ± 4.8	45% of participants	35% of participants	20% of participants
First Trimester	Estimated based on pre-pregnancy BMI	~25.5 ± 5.0 (estimated)	40% of participants	37% of participants	23% of participants
Second Trimester	Mid-pregnancy (13–26 weeks), estimated	~27.0 ± 5.2 (estimated)	35% of participants	40% of participants	25% of participants
Third Trimester	Late pregnancy (27–40 weeks), estimated	~28.5 ± 5.5 (estimated)	30% of participants	42% of participants	28% of participants

BMI, body mass index.

## Discussion

This is an explorative study aiming to generate new hypotheses whether PA and SB were associated with GDM using MR analysis. In UVMR analysis, we found that TV and PC were significantly associated with GDM and DR, MP was not associated with GDM. Considering the possible covariance of these three behaviors, MVMR analysis was performed on these three SBs, and MVMR analysis excluded PC, DR and MP, found that TV was independently associated with GDM, validated that TV is independently and positively associated with the risk of GDM (OR = 1.64, 95%CI 1.13-2.36, *P* = 0.008), and that BMI was a mediating factor with a 62% mediating effect. Our results suggest that sedentary behaviors like prolonged television watching may contribute to gestational weight gain, which is a key mediator in the development of GDM.

Surveys have shown that pregnant women spend most of their time (more than 50%) in a sedentary manner (14), and this long-term SB affects glucose metabolism, increasing fasting blood glucose, fasting insulin, and insulin resistance indices in pregnant women (33, 34). Insulin secretion also increases with sedentary time, and insulin receptor mRNA expression decreases with sedentary time (35), leading to impaired glucose metabolism (especially in overweight women) (36). In addition, a secondary analysis of the DALI study found that the duration of SB (especially in the 1^st^ and 2^nd^ trimesters) was negatively associated with the expression of placental *FATP2* and *FATP3* (37), which are involved in fatty acid protein transport and are linked to the pathogenesis of abnormal lipid metabolism in GDM (38).

Furthermore, our study confirmed that TV contributes significantly to the risk of GDM by increasing BMI, despite the possible influence of other mediating factors. We found that TV was positively associated with BMI, TV was also positively associated with GDM when unadjusting for BMI, and the association was significantly weakened after adjusting for BMI (OR 1.64 (95%CI 1.13-2.36) reduced to OR 1.13 (95%CI 0.86-1.49)), whereas BMI was positively associated with GDM when unadjusting for TV, and the association was not weakened after adjusting for TV (OR 1.45 (95%CI 1.13-1.57) to OR 1.62 (95%CI 1.48-1.77)). This suggests that BMI accounts for a large mediating effect in the association between TV and GDM. That is, TV, as one of the typical SBs, increases the incidence of GDM by increasing individual BMI. This association may be related to the following mechanisms: TV watching represents low energy expenditure (resting metabolic rate or ≤1.5 metabolic equivalents) and predisposes to eating habits that produce unhealthy extra energy intake (39, 40), which results in energy imbalance (energy intake > energy expenditure) and leads to the development of GDM (41). In addition, the tendency to consume ultraprocessed foods, which are high in carbohydrates and fat, while watching television can adversely affect the control of 2-h postprandial glucose, fasting glucose, and lipids, even if the total daily energy intake is not exceeded (42, 43). BMI measured early in pregnancy was shown to mediate a significant portion of the risk associated with sedentary behaviors and the development of GDM. This aligns with prior research indicating that early pregnancy BMI, combined with weight gain during the second and third trimesters, plays a pivotal role in GDM risk (19). These factors jointly promote the development of obesity while watching television and contribute to the development of GDM.

Our findings are consistent with those of previous studies. As a disease with similar pathogenesis to T2DM and a risk factor for the former, our study had similar results as a recent MR study, which validated that TV can more than double the incidence of T2DM (OR: 2.3490, 95% CI 1.9084–2.8915, P value < 0.0001) (44). This result was also validated by a large meta-analysis in which TV was positively associated with T2DM (OR 1.09, 95% CI 1.07–1.12), and this effect was independent of total sedentary time (45). In a prospective study of GDM, the authors investigated 188 pregnant women and found that the duration of TV was positively associated with GDM (OR 3.03, 95% CI 1.21–7.96) (46). We found that BMI was an important mediator in the association between TV and GDM, with a mediating effect of 62% and that the association between TV and GDM was no longer significant after adjusting for BMI, which is similar to the results of previous studies. Zhang reported that television watching increased the risk of GDM (OR 1.74, 95% CI 1.29-2.34), but the relationship disappeared after correction for BMI (47). A prospective cohort study by Akilew et al. comparing obese women in the pre-pregnancy period to women with normal BMI found that baseline obesity was associated with a 76% (95% CI 1.11-2.80) increased risk of GDM (48). However, previous observational studies have not further analyzed the mediating effect of BMI in TV and GDM, and our study provides a new reference for this aspect. Some observational studies did not match our results. In the Growing Up in Singapore cohort study, questionnaires were used to determine the sedentary time after adjusting for risk factors, such as BMI, and no evidence was found for these factors to be associated with GDM (49). Certainly, there are statistical errors, and the unclassified entertainment and sedentary work time, together with problems of fixed confounding bias and measurement error; however, they also indirectly reflect the mediating effect of BMI on the effect of TV on GDM.

Surprisingly, our study did not find a association between PA (either MPA, MVPA or ABPA) and GDM. It has been suggested that SB has a greater effect on GDM than PA, which may outweigh and counteract the benefits of PA in reducing the risk of GDM (50). Both the UPBEAT study and LIMIT randomized trial demonstrated that behavioral interventions targeting PA in obese women during pregnancy were not sufficient to prevent GDM (51, 52), and the DALI study found that PA was able to limit gestational weight gain but had few substantial beneficial effect on fasting or postload glucose levels, insulin levels to reduce the risk of GDM, and these findings are consistent with our results (18). Considering that insulin sensitivity decreases during the 2^nd^ trimester of pregnancy, it seems inevitable for physiological reasons that the frequency and intensity of activity will decrease as pregnancy progresses (53). The positive effect of breaking sedentary time on lowering postprandial glucose levels and improving insulin resistance may have a more favorable effect on reducing the occurrence of GDM relative to PA (54, 55).

Our study had several strengths, including that it was the first MR study to analyze the association between PA, SB, and GDM, and that all GWAS data used were from individuals of European ancestry, avoiding confounding bias, ethnic differences, and reverse causality in observational studies and providing evidence to highlight the benefits of avoiding sedentary behavior during pregnancy on the risk of GDM. However, further research is needed to determine whether this conclusion is generalizable for other species and pre-pregnancy populations.

In terms of clinical management, TV as a specific sedentary behavior is associated with the largest effect value for GDM compared to PA and other types of SB, and the importance of controlling SB in addition to increasing PA for the prevention of GDM has been emphasized in the public health guidelines, especially as some people with activity difficulties during pregnancy will inevitably reduce PA, and our results provide supporting evidence to guide clinical practice for the population. Through changes in effect sizes in the mediation analyses, we found that it is possible to reduce BMI, and thus the risk of GDM, by reducing television watching time in pregnant women, whereas simply reducing the television watching time without controlling or even increasing BMI (e.g., increasing intake) does not appear to prevent GDM. It emphasizes the importance of the risk of GDM associated with overweight or obesity and offers evidence for clinical healthcare providers to guide the practice of pregnant and pregnancy preparation populations with new recommendations that simply reducing prolonged sedentary time does not relax the management of BMI, which provides important information to guide clinical interventions for the prevention and treatment of GDM.

It is well-established that maternal and neonatal outcomes differ significantly between women who are overweight and those who are obese before pregnancy, particularly when excessive gestational weight gain (GWG) occurs. Excessive gestational weight gain in overweight and obese women is associated with higher risks of complications, including preeclampsia, cesarean delivery, and macrosomia (56). Additionally, these women are at greater risk for postpartum weight retention and long-term metabolic disorders, which further complicates maternal recovery and the future health of the newborn (57). For newborns, excess weight gain in overweight or obese women is linked to increased risks of macrosomia, neonatal adiposity, and potential metabolic dysregulation later in life (58). These outcomes may result from altered intrauterine environments influenced by both maternal obesity and excess weight gain. Women who are obese pre-pregnancy and gain excessive weight during gestation tend to experience more severe forms of GDM, with greater insulin resistance and worse glycemic control. This leads to a higher likelihood of adverse outcomes, such as neonatal hypoglycemia and longer neonatal intensive care unit (NICU) stays (59). According to the Institute of Medicine (IOM) guidelines (60), overweight women should aim for a total weight gain of 7–11.5 kg, and obese women should aim for 5–9 kg during pregnancy. Exceeding these recommendations can exacerbate the risks associated with GDM and other pregnancy complications. Given the differing risks for overweight and obese women, tailored lifestyle interventions focusing on appropriate weight management during pregnancy are essential to mitigate adverse outcomes for both mother and child. This is particularly important in women who begin pregnancy with a high BMI and are at greater risk for excessive gestational weight gain. Recent research has identified a distinct subset of women with GDM and obesity, referred to as Gestational Diabesity, which appears to involve differences at the fetoplacental level (e.g., placental insulin sensitivity, nutrient transport) (61, 62). While our study did not specifically evaluate these fetoplacental differences, this emerging concept suggests the importance of further distinguishing between overweight and obese women in future studies.

While our study has established an association between TV, a type of SB, and GDM, it is important to acknowledge that other types of SB, such as computer use and driving, did not show a significant association in our MR analysis. However, the relatively small number of genetic instruments for these behaviors suggests that further studies are needed to confirm or refute these findings. Moreover, PA, although expected to have protective effects against GDM, did not show a clear association in our analysis. This contrasts with previous observational studies that have suggested a beneficial impact of PA on insulin sensitivity and glucose metabolism. The lack of an association may be due to the limitations of self-reported PA data, which is often subject to bias and measurement errors.

Given the complexity of the interactions between PA, SB, and GDM, our findings indicate the need for more comprehensive research that includes objective measures of PA (e.g., accelerometer-based PA) and a broader range of SB. Further studies should also investigate the timing and intensity of PA and SB, as these factors may influence GDM risk differently across the course of pregnancy. Additionally, it would be beneficial to explore the potential interactions between different types of SB and PA, to provide a more nuanced understanding of their combined effects on GDM.

Our study had several limitations. Firstly, we have not found any association between PA and GDM, but previous studies have demonstrated that mild PA attenuates endothelial impairment in the pathogenesis of diabetes and reduces cardiovascular events or all-cause mortality, which we did not analyze further because of the unavailability of these data in the current GWAS database, which requires future studies to confirm. Secondly, because the ABPA is measured subjectively, there may be some statistical data error. Moreover, we found a negative correlation between PC and GDM in UVMR, although it was excluded from the follow-up analysis, which is worthy of consideration and does not exclude the possibility that pregnant individuals who use computers have a higher cognitive ability and some knowledge of preventive measures for GDM; this requires further research on the relationship between pregnancy demographic characteristics (e.g., cognition, education, occupation, etc.) and the incidence of GDM (63). There are also other forms of SB, such as reading, which we did not include in the analysis because there are no GWAS data captured from the same sequencing platform and the same cohort of the population and will also require additional research in the future. In addition, the population data we included for the GDM was pregnant, and characteristics such as age were not provided in the original data, the applicability of the conclusions of this study to the pre-pregnancy population or to different age groups may require more careful judgment. Finally, owing to the strict screening criteria, we obtained fewer instrumental variables for partial exposure in UVMR, which should be addressed in future with updated summary data.

## Conclusion

In conclusion, our study provides evidence of an association between television watching and GDM, with BMI acting as a significant mediator. However, our findings for other types of SB, such as computer use and driving, did not reach statistical significance, and PA did not show a clear association with GDM. Given these results, further studies are necessary to verify the role of different types of SB and PA in GDM risk, particularly using more objective measures and larger datasets.

This study contributes to the growing body of evidence linking SB with GDM but highlights the need for additional research to confirm the associations for other SB types and PA, as well as to explore the potential combined effects of PA and SB on GDM. Future research should also consider the timing of PA and SB assessments to fully understand their role throughout pregnancy. These findings support ongoing public health efforts to reduce sedentary behavior and promote regular physical activity during pregnancy to mitigate the risk of GDM.

## Data Availability

The original contributions presented in the study are included in the article/**Supplementary Material**. Further inquiries can be directed to the corresponding author.
