# Supplementary material for: Association between physical activity and sedentary behavior and gestational diabetes mellitus: a Mendelian randomization analysis
Source: Front Endocrinol (Lausanne). 2024 Dec 16;15:1389453. doi: 10.3389/fendo.2024.1389453 (PMC11682963; doi:10.3389/fendo.2024.1389453)
Supplement: Supplementary file 1 [file SupplementaryFile1.pdf]

Supplementary material

Table S1. Characteristics of the genetic instruments associated with exposure

Figure S1. Scatter plot of SNPs effect sizes with MR-PRESSO outlier removed

Figure S2. Leave-one-out analysis with MR-PRESSO outlier removed

Supplementary Table S1 Characteristics of the genetic instruments associated with exposure

Exposure		SNP	Gene	Effect allele	Other allele	Beta	SE	EAF	Pvalue	R^2	F-statistics
		rs1538360	RP11	G	A	-0.012	0.002	0.553	3.80E-09	7.12E-05	24.479
	MPA	rs682245	TCF4	C	T	0.012	0.002	0.524	2.30E-09	7.18E-05	24.700
		rs7222403	AKAP10	C	T	0.011	0.002	0.426	3.60E-08	5.92E-05	20.347
		rs7610133	LRRN1	C	T	-0.013	0.002	0.260	4.70E-08	6.50E-05	22.361
		rs10145335	CADM2	A	G	0.014	0.003	0.251	2.7E-08	7.49E-05	28.260
		rs1043595	HIST1H4(	A	G	-0.014	0.002	0.283	4.30E-09	8.43E-05	31.787
		rs1186721	EXOC4	A	G	0.013	0.002	0.316	4.40E-08	7.29E-05	27.512
		rs12912808	FOXO3	T	C	-0.018	0.003	0.149	1.70E-08	7.79E-05	29.390
		rs1921981	DPY19L1	A	G	-0.013	0.002	0.326	3.80E-08	7.46E-05	28.162
		rs1972763	CTBP2	T	C	-0.013	0.002	0.658	3.30E-08	7.42E-05	28.000
		rs1974771	ACYP2	A	G	0.021	0.004	0.100	6.60E-09	8.19E-05	30.915
		rs2035562	CADM2	G	A	0.014	0.002	0.672	3.90E-09	8.48E-05	31.999
		rs2114286	RP11	G	A	0.012	0.002	0.534	3.30E-08	7.46E-05	28.152
		rs2942127	PIK3C2I	A	G	-0.016	0.003	0.825	3.30E-08	7.44E-05	28.061
	MVPA	rs2988004	RP11	G	T	0.013	0.002	0.442	4.10E-09	8.56E-05	32.285
PA		rs429358	APOE	C	T	0.022	0.003	0.154	6.10E-13	1.26E-04	47.547
		rs4856278	CADM2	G	A	-0.015	0.003	0.758	1.70E-08	7.92E-05	29.883
		rs4886868	DNM1P	G	T	0.012	0.002	0.586	3.50E-08	7.58E-05	28.583
		rs7326482	RP11	T	G	0.013	0.002	0.615	1.60E-08	7.95E-05	30.004
		rs77742115	CTD	C	T	0.018	0.003	0.138	9.60E-09	8.02E-05	30.275
		rs7804463	EXOC4	C	T	-0.015	0.002	0.470	1.20E-11	1.12E-04	42.348
		rs877483	CACNA	C	T	-0.012	0.002	0.567	4.00E-08	7.34E-05	27.700
		rs921915	AC0207	C	T	0.014	0.002	0.588	5.70E-10	9.35E-05	35.259
		rs9266639	HAX1	T	C	-0.014	0.002	0.281	8.50E-09	8.05E-05	30.358
		rs9272302	TBC1D22	T	C	-0.018	0.003	0.166	3.40E-09	9.20E-05	34.714
		rs11012732	MLLT10	G	A	-0.225	0.039	0.332	5.4E-09	2.25E-02	2092.569
		rs12522261	LINC0147	A	G	-0.211	0.038	0.343	3.90E-08	2.00E-02	1857.682
	ABPA	rs148193266	RP11	C	A	0.510	0.092	0.043	3.10E-08	2.13E-02	1980.274
		rs34517439	DNAJB4	A	C	-0.308	0.056	0.121	4.40E-08	2.02E-02	1873.982
		rs56194509	LINC0221	G	T	0.303	0.044	0.220	5.00E-12	3.16E-02	2976.286
		rs9293503	LINC0046	C	T	-0.329	0.059	0.112	2.10E-08	2.14E-02	1995.688
		rs10041445	AC010376.	C	T	-0.009	0.002	0.497	8.7E-09	4.19E-05	18.346
		rs10189857	BCL11A	G	A	0.015	0.002	0.432	5.70E-20	1.06E-04	46.362
		rs10269099	GRM8	T	G	0.009	0.002	0.391	2.10E-08	3.99E-05	17.459
		rs10765776	NA	A	C	-0.011	0.002	0.391	4.80E-12	6.10E-05	26.729
		rs11191129	C10orf76	T	C	-0.010	0.002	0.423	9.30E-10	4.73E-05	20.722
		rs11222919	NTM	G	T	-0.012	0.002	0.175	6.30E-09	4.31E-05	18.895
		rs11242455	SIL1	T	C	0.010	0.002	0.727	2.90E-08	4.00E-05	17.524
		rs11245482	CTBP2	C	T	0.010	0.002	0.385	4.80E-10	4.92E-05	21.537
		rs114755463	LINC0147	A	G	0.013	0.002	0.168	6.70E-10	4.91E-05	21.495
		rs115608101	RP11	T	C	-0.014	0.002	0.130	1.10E-08	4.16E-05	18.198
		rs11662211	KCNG2	T	C	-0.009	0.002	0.506	2.90E-09	4.46E-05	19.542
		rs11680095	UBE2E3	T	C	-0.009	0.002	0.593	1.90E-08	4.08E-05	17.855
		rs11696187	MIR646H	T	C	-0.014	0.002	0.160	4.20E-10	4.95E-05	21.686
		rs11700249	BTBD3	G	T	0.009	0.002	0.409	5.90E-09	4.30E-05	18.834
		rs11714337	FOXP1	A	G	-0.010	0.002	0.430	7.60E-10	4.82E-05	21.098
		rs11877758	CELF4	G	T	0.011	0.002	0.313	1.30E-10	5.27E-05	23.081
		rs11938781	LCORL	C	T	0.013	0.002	0.166	2.00E-09	4.58E-05	20.059
		rs12045585	AKT3	A	G	-0.015	0.002	0.131	4.10E-10	5.17E-05	22.651
		rs12192804	RP11	C	A	-0.016	0.003	0.095	4.70E-09	4.35E-05	19.039
		rs12725934	DPYD	A	T	-0.012	0.002	0.261	3.20E-10	5.20E-05	22.778
		rs1291871	CELF2	C	T	0.009	0.002	0.514	9.90E-09	4.19E-05	18.366
		rs13014947	PCGEM1	A	G	0.011	0.002	0.575	6.50E-11	5.50E-05	24.085
		rs13107325	SLC39A8	T	C	0.020	0.003	0.075	1.70E-11	5.74E-05	25.118
		rs1324491	CYP2J2	A	G	0.013	0.002	0.131	1.30E-08	4.10E-05	17.934
		rs1451533	LINC0115	A	G	0.010	0.002	0.274	4.40E-08	3.84E-05	16.823
		rs147067	DIAPH3	A	G	0.010	0.002	0.755	2.10E-08	4.00E-05	17.502
		rs1563908	NDUFAF	G	A	-0.010	0.002	0.378	4.00E-10	4.96E-05	21.706
		rs1727332	C12orf65	T	C	0.013	0.002	0.754	1.10E-12	6.42E-05	28.128
		rs1730858	PRMT6	C	T	-0.009	0.002	0.651	2.90E-08	3.97E-05	17.386
		rs17789218	RP3	C	T	-0.011	0.002	0.244	3.60E-09	4.40E-05	19.248
		rs178203	NOVA1	C	T	0.012	0.002	0.750	5.90E-11	5.42E-05	23.731
		rs1826510	CTD	A	G	-0.013	0.002	0.828	1.90E-08	4.61E-05	20.178
		rs204092	MACROD	C	T	-0.010	0.002	0.624	9.00E-09	4.26E-05	18.634
		rs2073869	SPACA9	T	C	-0.014	0.002	0.167	1.10E-10	5.29E-05	23.161

	rs2106164	RN7SL7P	C	T	-0.009	0.002	0.532	4.20E-09	4.39E-05	19.225
	rs2185490	GALNT16	C	A	0.009	0.002	0.618	1.20E-08	4.15E-05	18.169
	rs2240857	GLCCI1	G	T	0.016	0.002	0.141	4.50E-12	6.15E-05	26.926
	rs2283	EFNA5	G	A	-0.009	0.002	0.339	3.20E-08	3.85E-05	16.870
	rs2352984	MON1A	C	T	0.020	0.002	0.431	7.40E-35	1.91E-04	83.848
	rs2479968	TEX29	G	A	0.021	0.004	0.051	2.70E-08	4.11E-05	18.008
	rs249960	CTD	G	A	-0.012	0.002	0.182	1.10E-08	4.15E-05	18.177
	rs2608990	DOCK3	T	C	0.016	0.002	0.126	2.50E-11	5.62E-05	24.619
	rs262890	AC010376	G	A	0.013	0.002	0.299	2.60E-14	7.35E-05	32.176
	rs263771	ZNF804A	A	C	0.012	0.002	0.233	1.90E-10	5.17E-05	22.621
	rs2646351	RP11	A	G	0.009	0.002	0.453	2.40E-08	3.94E-05	17.244
	rs2678662	AC013727	G	T	0.011	0.002	0.608	8.6E-12	5.92E-05	25.924
	rs2725371	PURG	G	A	-0.013	0.002	0.696	2.1E-14	7.45E-05	32.628
	rs2857693	PRRC2A	T	G	0.011	0.002	0.366	9.60E-11	5.27E-05	23.072
	rs3138499	GADD45G	C	A	0.011	0.002	0.518	8.50E-12	6.00E-05	26.285
	rs319068	RP11	C	T	-0.009	0.002	0.549	2.60E-08	3.93E-05	17.192
	rs34094119	NA	G	A	0.009	0.002	0.525	1.30E-08	4.11E-05	18.014
	rs34109383	RP11	T	C	0.010	0.002	0.327	8.00E-09	4.21E-05	18.436
	rs34811474	ANAPC4	A	G	-0.013	0.002	0.231	3.10E-12	6.14E-05	26.904
	rs34819186	TSNARE1	C	A	0.009	0.002	0.473	3.30E-08	3.88E-05	17.003
	rs34864022	LINC0123	G	A	0.021	0.003	0.065	4.60E-11	5.53E-05	24.212
	rs35797019	RNU6-461	G	A	-0.009	0.002	0.392	1.5E-08	4.05E-05	17.728
	rs362312	HTT	C	T	-0.010	0.002	0.424	3.5E-09	4.43E-05	19.405
	rs3754970	TANK	C	T	0.010	0.002	0.503	1.3E-09	4.71E-05	20.607
	rs38004	GLCCI1	T	C	-0.009	0.002	0.456	6.8E-09	4.25E-05	18.628
	rs3810496	ZBTB46	C	T	0.009	0.002	0.616	1.2E-08	4.15E-05	18.190
	rs4076457	LINGO1	T	C	-0.010	0.002	0.257	3.7E-08	3.85E-05	16.848
TV	rs4110177	CTD	A	G	0.009	0.002	0.367	1.3E-08	4.13E-05	18.088
	rs4303732	LINC0110	C	T	-0.011	0.002	0.402	3.7E-11	5.53E-05	24.223
	rs4339469	RP11	G	T	0.013	0.002	0.629	2.8E-14	7.32E-05	32.075
	rs44205	OVOL1	A	G	-0.009	0.002	0.331	2.9E-08	3.88E-05	16.989
	rs4469687	EDEM3	G	A	0.009	0.002	0.484	3.1E-08	3.87E-05	16.933
	rs4554203	HTR4	A	G	-0.010	0.002	0.420	9.9E-10	4.73E-05	20.718
	rs4675246	RP11	T	G	0.012	0.002	0.200	2.1E-09	4.51E-05	19.736
	rs4688759	RBM6	C	T	-0.016	0.003	0.106	8.1E-10	4.77E-05	20.884
	rs4740589	RNA5SP2	G	A	-0.009	0.002	0.348	3.2E-08	4.07E-05	17.831
	rs4747438	DNAJC1	T	C	-0.012	0.002	0.677	3.9E-12	6.11E-05	26.776
	rs4788616	PMFBP1	G	T	-0.010	0.002	0.391	4.3E-10	4.94E-05	21.628
	rs4911257	DNMT3B	C	T	0.010	0.002	0.393	2.3E-09	4.53E-05	19.857
	rs494566	RP11	T	C	0.010	0.002	0.332	2E-09	4.58E-05	20.075
	rs507021	DMRTA1	T	C	0.009	0.002	0.571	4.3E-08	3.80E-05	16.661
	rs56398417	HSPE1P19	T	C	-0.010	0.002	0.311	1.3E-08	4.09E-05	17.907
	rs60737686	HSPE1P19	G	A	-0.012	0.002	0.156	3E-08	3.93E-05	17.217
	rs6102912	PTPRT	C	T	-0.011	0.002	0.410	1.4E-11	5.76E-05	25.212
	rs61743199	NA	G	A	0.017	0.003	0.072	1.6E-08	4.03E-05	17.648
	rs61864793	HMGN2P	C	T	-0.011	0.002	0.252	1.2E-09	4.68E-05	20.477
	rs62145951	PNO1	C	T	-0.012	0.002	0.263	2.6E-11	5.61E-05	24.552
	rs62199883	VWC2L	A	C	0.014	0.002	0.486	3.2E-18	9.57E-05	41.925
	rs6511708	ILF3	C	T	-0.012	0.002	0.665	7.5E-13	6.51E-05	28.491
	rs6740081	DPP10	C	T	0.011	0.002	0.711	2.1E-09	4.54E-05	19.893
	rs68056254	AC062032	T	G	0.013	0.002	0.151	1.3E-09	4.67E-05	20.434
	rs6814554	FAM160A	A	G	0.014	0.002	0.474	2.4E-17	9.13E-05	39.964
	rs6850494	RP11	C	A	0.010	0.002	0.386	3.4E-09	4.42E-05	19.370
	rs6876982	RP11	T	C	-0.010	0.002	0.255	4.3E-08	3.80E-05	16.644
	rs6895658	LMNB1	C	T	-0.013	0.002	0.193	1.1E-10	5.25E-05	23.009
	rs6920115	LINC0024	C	T	-0.018	0.003	0.093	2.2E-11	5.65E-05	24.752
	rs6929983	TTBK1	T	C	0.012	0.002	0.166	2.7E-08	3.92E-05	17.147
	rs6994132	RP11	C	T	-0.010	0.002	0.579	6.2E-10	4.84E-05	21.188
	rs7089973	TAF9BP2	A	C	0.009	0.002	0.380	1.6E-08	4.07E-05	17.831
	rs72768080	WDR64	C	T	-0.012	0.002	0.210	2.1E-09	4.69E-05	20.556
	rs727809	CTB	C	A	-0.009	0.002	0.431	6.9E-09	4.27E-05	18.681
	rs73077107	BSN	A	G	-0.019	0.002	0.125	2.4E-15	7.90E-05	34.612
	rs73560982	RP1	C	T	-0.019	0.003	0.060	2.5E-08	3.92E-05	17.183
	rs73571431	CRB2	T	C	0.016	0.003	0.110	5.5E-10	4.90E-05	21.469
	rs73946726	RP11	A	C	0.032	0.006	0.020	2.2E-08	4.05E-05	17.739
	rs7414210	DPYD	C	A	0.013	0.002	0.161	4.4E-09	4.40E-05	19.289
	rs749056	CYB561D	G	T	-0.010	0.002	0.304	7.2E-09	4.25E-05	18.615
	rs749671	ZNF646	A	G	-0.011	0.002	0.372	1.6E-11	5.74E-05	25.119
	rs7539775	PRDM16	A	G	0.010	0.002	0.743	4.3E-08	3.79E-05	16.602
	rs75499503	ZFP57	T	C	-0.018	0.002	0.220	3.1E-21	1.16E-04	50.990

	rs75641275	DPYD	C	A	0.015	0.002	0.143	1E-11	5.85E-05	25.614
	rs76608582	CTB	A	C	-0.022	0.004	0.047	3.4E-08	4.26E-05	18.638
	rs77779142	SNX32	T	C	0.012	0.002	0.166	2.6E-08	3.91E-05	17.129
	rs7798292	RP11	A	G	-0.010	0.002	0.435	1.3E-09	4.64E-05	20.340
	rs78227853	FRMD5	T	C	-0.028	0.005	0.025	3.4E-08	3.89E-05	17.028
	rs7899206	RPS27P18	G	T	-0.010	0.002	0.492	1.3E-09	4.78E-05	20.919
	rs7921305	PPP2R2D	A	G	-0.011	0.002	0.253	3.7E-10	4.97E-05	21.762
	rs79373894	NEO1	C	T	-0.029	0.004	0.034	9.8E-11	5.48E-05	23.999
	rs801733	PACS1	C	A	-0.012	0.002	0.358	5E-14	7.17E-05	31.407
	rs814197	LINC0174	G	T	-0.011	0.002	0.467	2E-11	5.68E-05	24.882
	rs830622	RP11	C	T	0.012	0.002	0.171	3.8E-09	4.40E-05	19.251
	rs898751	MNT	T	C	0.010	0.002	0.493	3E-10	5.02E-05	21.985
	rs909892	FTLP1	A	G	-0.014	0.002	0.135	9E-10	4.79E-05	20.979
	rs9300594	PCCA	G	A	0.011	0.002	0.254	9.1E-10	4.75E-05	20.803
	rs9471333	LRFN2	T	C	-0.010	0.002	0.552	6.7E-11	5.38E-05	23.546
	rs9834970	LINC0203	C	T	-0.009	0.002	0.498	2E-08	3.97E-05	17.375
	rs9867121	ZBTB20	A	C	-0.012	0.002	0.184	1.4E-08	4.11E-05	18.007
	rs9867437	CADM2	C	A	-0.010	0.002	0.460	1.3E-10	5.28E-05	23.103
SB	rs996234	PDE4D	A	G	-0.011	0.002	0.516	1.6E-10	5.55E-05	24.308
	rs10208088	AC11476	T	C	-0.010	0.002	0.580	3E-08	5.11E-05	18.448
	rs1037091	KCNJ3	T	C	-0.015	0.002	0.327	5.9E-15	1.05E-04	38.075
	rs10518019	AC112518	G	A	0.010	0.002	0.476	4.9E-08	4.93E-05	17.802
	rs10828248	MLLT10	G	A	0.011	0.002	0.345	2.8E-08	5.13E-05	18.505
	rs11259902	RP11	A	C	0.013	0.002	0.200	1.6E-08	5.34E-05	19.273
	rs112600282	LINC0187	G	A	-0.017	0.003	0.110	7.2E-09	5.62E-05	20.285
	rs113851275	PTCH1	A	G	0.019	0.003	0.108	2.5E-10	6.66E-05	24.030
	rs11576509	SORT1	T	C	0.033	0.005	0.030	2.1E-09	6.14E-05	22.152
	rs11634155	AC009878	C	T	-0.012	0.002	0.335	2.5E-09	6.01E-05	21.702
	rs11652437	RP11	A	C	0.013	0.002	0.324	1E-10	7.15E-05	25.823
	rs11749912	MEF2C	G	A	-0.013	0.002	0.575	3.7E-12	8.11E-05	29.285
	rs11942953	LINC0217	C	T	-0.010	0.002	0.537	1.3E-08	5.44E-05	19.623
	rs12128707	NEGR1	G	A	0.012	0.002	0.264	7.1E-09	5.63E-05	20.313
	rs12145677	SYPL2	A	G	0.016	0.002	0.297	6.4E-15	1.01E-04	36.594
	rs1229984	ADH1B	C	T	-0.034	0.006	0.973	4.4E-10	6.32E-05	22.799
	rs12521638	HMP19	G	A	0.010	0.002	0.447	4.2E-08	5.02E-05	18.119
	rs12706626	POT1	A	G	0.010	0.002	0.384	2.9E-08	5.12E-05	18.495
	rs12820967	RP11	C	T	0.012	0.002	0.322	1.5E-09	6.18E-05	22.298
	rs13262595	TSNARE1	G	A	0.016	0.002	0.561	1.8E-17	1.20E-04	43.449
	rs13422733	RFX8	T	C	-0.015	0.003	0.126	4.1E-08	5.04E-05	18.190
	rs136553	LINC0142	T	C	0.011	0.002	0.377	2E-09	6.01E-05	21.678
	rs1395020	RP11	A	G	-0.011	0.002	0.303	3.4E-08	5.11E-05	18.431
	rs1448355	NTM	T	C	0.012	0.002	0.618	8E-11	7.09E-05	25.606
	rs1469249	RP11	A	G	-0.013	0.002	0.211	8E-09	5.60E-05	20.198
	rs147543875	DNMBP	T	C	-0.036	0.006	0.023	1.5E-08	5.85E-05	21.099
	rs1524909	RNU6-546	G	A	0.011	0.002	0.342	3.7E-08	5.05E-05	18.215
	rs1648906	RPL12P40	A	G	-0.011	0.002	0.321	2.1E-08	5.32E-05	19.183
	rs166835	SEMA6D	T	C	-0.011	0.002	0.556	4.3E-09	5.78E-05	20.871
	rs16912540	ARNTL	G	A	-0.016	0.003	0.137	2.7E-09	5.91E-05	21.322
	rs17167210	EXOC4	A	G	-0.011	0.002	0.436	6E-10	6.39E-05	23.052
	rs17789218	RP3	C	T	0.013	0.002	0.245	2.1E-09	5.97E-05	21.529
	rs17862355	ZNF800	G	T	-0.011	0.002	0.442	1E-09	6.20E-05	22.375
	rs1987942	RP11	C	T	-0.011	0.002	0.617	5.4E-09	5.78E-05	20.861
	rs2032780	SPAG16	C	T	0.012	0.002	0.406	4.5E-11	7.36E-05	26.550
	rs2041687	RP11	G	T	0.012	0.002	0.549	3.8E-10	6.79E-05	24.503
	rs2068625	C4orf45	C	T	0.014	0.002	0.698	1E-12	8.46E-05	30.547
	rs206965	COX6A1	C	T	-0.013	0.002	0.791	7.9E-09	5.54E-05	19.984
	rs2120461	RERE	T	C	0.012	0.002	0.661	2.5E-10	6.65E-05	24.010
	rs2307022	PRMT7	G	A	-0.011	0.002	0.668	8.3E-09	5.52E-05	19.910
	rs246723	PCDHB5	G	A	-0.011	0.002	0.590	1.2E-08	5.50E-05	19.866
	rs2588543	RP11	T	C	0.011	0.002	0.672	2.2E-08	5.25E-05	18.931
	rs2734833	DRD2	A	G	0.012	0.002	0.607	3.8E-11	7.31E-05	26.379
PC	rs2748985	TMEM52	C	T	0.012	0.002	0.544	9.4E-12	7.75E-05	27.961
	rs2761438	KCNC4	G	A	-0.011	0.002	0.624	1.7E-09	6.03E-05	21.764
	rs28710456	GATB	C	T	-0.010	0.002	0.490	1.2E-08	5.41E-05	19.532
	rs306755	UBOX5	C	T	0.010	0.002	0.475	1.4E-08	5.37E-05	19.367
	rs34179846	DYNLL1	C	T	-0.017	0.003	0.105	1E-08	5.45E-05	19.687
	rs34238696	LINC0120	G	A	-0.017	0.003	0.107	1.3E-08	5.39E-05	19.468
	rs3730399	E2F4	G	A	-0.023	0.004	0.064	9.1E-10	6.26E-05	22.590
	rs4704043	TNPO1	T	C	0.012	0.002	0.714	8.9E-09	5.51E-05	19.878
	rs4852252	ZNF638	C	T	0.010	0.002	0.564	3E-08	5.11E-05	18.443

	rs56229818	ALDH1A2	C	T	-0.010	0.002	0.484	3E-08	5.12E-05	18.461
	rs585282	AUTS2	T	C	0.011	0.002	0.648	2.8E-09	5.90E-05	21.292
	rs58638214	NA	T	C	-0.013	0.002	0.398	6.7E-12	7.89E-05	28.465
	rs6028090	CDH4	A	G	0.013	0.002	0.555	1.3E-11	7.76E-05	28.009
	rs613872	TCF4	T	G	-0.016	0.002	0.827	7.7E-11	7.07E-05	25.528
	rs62553704	RP11	T	C	0.023	0.004	0.058	7.7E-09	5.67E-05	20.472
	rs627685	TCF4	C	T	-0.013	0.002	0.304	3.2E-10	6.69E-05	24.138
	rs6449708	AC11660[illegible]	C	T	-0.011	0.002	0.532	7.9E-09	5.57E-05	20.108
	rs6780848	LMCD1-[illegible]	G	T	0.011	0.002	0.270	3.3E-08	5.09E-05	18.383
	rs6935828	AL35673[illegible]	T	C	0.010	0.002	0.556	3.1E-08	5.10E-05	18.417
	rs7020477	AMBP	G	A	-0.012	0.002	0.267	7E-09	5.61E-05	20.250
	rs707926	VARS	A	G	0.015	0.003	0.150	1.2E-08	5.40E-05	19.498
	rs7209653	AKAP10	C	T	-0.013	0.002	0.295	2.2E-11	7.46E-05	26.930
	rs7221299	B9D1	A	G	-0.019	0.003	0.107	3.5E-10	6.55E-05	23.643
	rs72828532	RP1	C	T	0.016	0.002	0.179	4.5E-11	7.24E-05	26.126
	rs72847500	MDGA1	C	T	0.016	0.003	0.121	1.9E-08	5.31E-05	19.181
	rs7288455	CACNA1[illegible]	G	A	-0.011	0.002	0.567	1.1E-08	5.46E-05	19.719
	rs7335281	MIR4704	G	A	-0.013	0.002	0.779	2.7E-09	5.91E-05	21.326
	rs73578186	DENND1[illegible]	T	C	-0.012	0.002	0.324	3.7E-10	6.62E-05	23.884
	rs7460106	ADGRB1	C	T	0.014	0.002	0.236	3.3E-10	6.77E-05	24.432
	rs7485383	C12orf40	A	G	-0.013	0.002	0.754	1.6E-09	6.04E-05	21.784
	rs7526112	CCDC18	G	T	-0.011	0.002	0.362	1.1E-08	5.43E-05	19.612
	rs75550998	AC07924[illegible]	T	G	-0.022	0.004	0.054	4.6E-08	5.08E-05	18.351
	rs7630869	DAG1	T	C	0.016	0.002	0.304	4E-16	1.10E-04	39.673
	rs7662509	RNU6-88[illegible]	A	G	0.011	0.002	0.428	6.6E-09	5.62E-05	20.289
	rs7663288	RP11	G	A	0.012	0.002	0.729	1.2E-08	5.45E-05	19.665
	rs7904398	CTNNA3	T	C	-0.010	0.002	0.503	2.4E-08	5.20E-05	18.774
	rs7968738	RP11	A	G	-0.012	0.002	0.264	2.4E-09	5.99E-05	21.615
	rs79720045	RP11	C	T	-0.013	0.002	0.401	1.8E-11	7.82E-05	28.235
	rs8046159	PKD1L3	G	A	0.010	0.002	0.518	1.7E-08	5.31E-05	19.151
	rs806795	HIST1H4[illegible]	A	G	0.010	0.002	0.470	1.4E-08	5.34E-05	19.284
	rs9288497	VWC2L	G	A	0.014	0.002	0.238	8.1E-11	7.03E-05	25.372
	rs9375188	RP11	T	C	0.016	0.002	0.484	5E-18	1.26E-04	45.301
	rs9537571	PRR20A	A	G	0.019	0.003	0.096	1.1E-09	6.21E-05	22.414
DR	rs11057408	ZNF664	T	G	-0.011	0.002	0.334	1.2E-08	5.69E-05	17.686
	rs12068763	RP11	G	T	0.012	0.002	0.219	3.7E-08	5.33E-05	16.562
	rs12921753	DOC2A	T	C	0.012	0.002	0.400	2.9E-10	6.96E-05	21.631
	rs2090660	AC06849[illegible]	T	C	-0.013	0.002	0.194	3.7E-08	5.35E-05	16.622
	rs2588917	C10orf10[illegible]	A	C	-0.010	0.002	0.448	3.4E-08	5.39E-05	16.734
	rs975303	RP1	G	A	0.015	0.002	0.181	1.3E-09	6.48E-05	20.117
MP	rs12145998	RP11	T	C	-0.019	0.003	0.266	2.9E-09	2.75E-05	12.553
	rs1892417	LINC018[illegible]	C	T	0.026	0.003	0.229	1.3E-14	4.86E-05	22.190
	rs344868	PTCH1	T	C	0.018	0.003	0.247	3.2E-08	3.34E-05	15.274
	rs6718176	SORT1	G	C	-0.018	0.003	0.513	1.7E-10	4.23E-05	19.353
	rs359265	AC00987[illegible]	A	G	0.021	0.003	0.607	6.4E-13	5.63E-05	25.719
	rs11682846	RP11	T	C	-0.017	0.003	0.485	9.9E-10	4.04E-05	18.444
	rs849527	MEF2C	G	A	-0.016	0.003	0.547	1.5E-08	7.77E-06	3.551
	rs6780051	LINC021[illegible]	T	G	0.040	0.006	0.058	6.9E-11	4.57E-05	20.882
	rs1512142	NEGR1	A	G	-0.017	0.003	0.443	8.3E-09	2.66E-05	12.151
	rs17374152	SYPL2	G	A	-0.019	0.003	0.239	5.3E-09	3.12E-05	14.274
	rs17156711	EDEM3	G	A	-0.018	0.003	0.299	4.1E-09	2.80E-05	12.774
	rs2161220	HTR4	A	G	0.021	0.003	0.248	4E-10	1.45E-05	6.627
	rs78166132	RP11	C	T	-0.031	0.005	0.094	4.9E-10	3.41E-05	15.591
	rs10807124	RBM6	A	G	-0.018	0.003	0.274	3.1E-08	3.08E-05	14.087
	rs28713780	RNA5SP2[illegible]	C	T	-0.017	0.003	0.641	1.1E-08	3.14E-05	14.347
	rs13266457	DNAJC1	T	C	-0.017	0.003	0.330	1.7E-08	3.47E-05	15.850
	rs10107145	PMFBP1	G	A	-0.018	0.003	0.545	1.7E-10	2.13E-05	9.740
	rs7859831	DNMT3B	T	C	-0.023	0.004	0.139	2.1E-08	3.12E-05	14.251
	rs10828247	RP11	G	A	0.017	0.003	0.344	7.4E-09	3.65E-05	16.694
	rs853946	RP11	T	C	0.016	0.003	0.468	1.8E-08	3.21E-05	14.670
	rs1320650	DNMBP	A	T	0.017	0.003	0.367	7.2E-09	2.31E-05	10.557
	rs11236714	RNU6-54[illegible]	T	C	-0.020	0.004	0.196	1.8E-08	7.92E-06	3.619
	rs11229008	RPL12P4[illegible]	A	G	-0.033	0.006	0.063	3.9E-08	3.38E-05	15.461
	rs8014346	SEMA6D	A	G	0.019	0.003	0.535	3.7E-11	3.93E-05	17.964
	rs12437348	ARNTL	A	G	0.017	0.003	0.710	4.2E-08	9.45E-06	4.316
	rs77878475	EXOC4	A	T	-0.032	0.005	0.084	2.1E-09	3.88E-05	17.716
	rs11655813	RP3	T	C	0.018	0.003	0.355	1.2E-09	4.03E-05	18.426
	rs9896202	ZNF800	C	T	-0.021	0.003	0.498	1.9E-13	5.63E-05	25.721
	rs6131703	RP11	G	A	-0.018	0.003	0.386	1.9E-09	2.71E-05	12.402
	rs6063374	SPAG16	G	A	0.029	0.003	0.781	1E-17	7.52E-05	34.368

	rs2836920	RP11	G	T	0.019	0.003	0.396	2.3E-10	3.56E-05	16.248

SNP single-nucleotide polymorphisms, SE standard error, EAF effect allele frequency

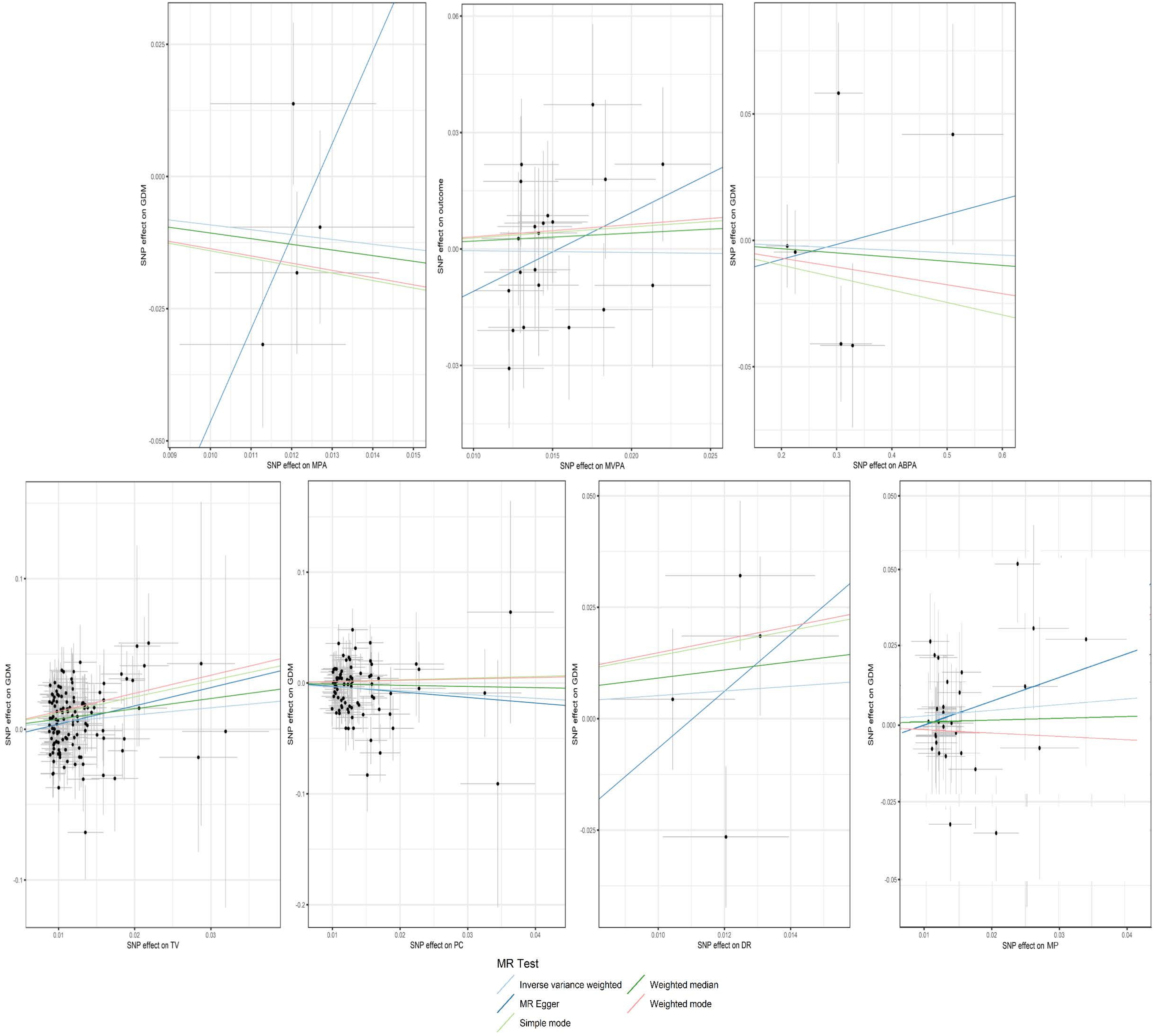
SNP effect on GDM
SNP effect on MPA
SNP effect on outcome
SNP effect on MVPA
SNP effect on ABPA
SNP effect on TV
SNP effect on PC
SNP effect on DR
SNP effect on MP
MR Test
Inverse variance weighted
MR Egger
Simple mode
Weighted median
Weighted mode

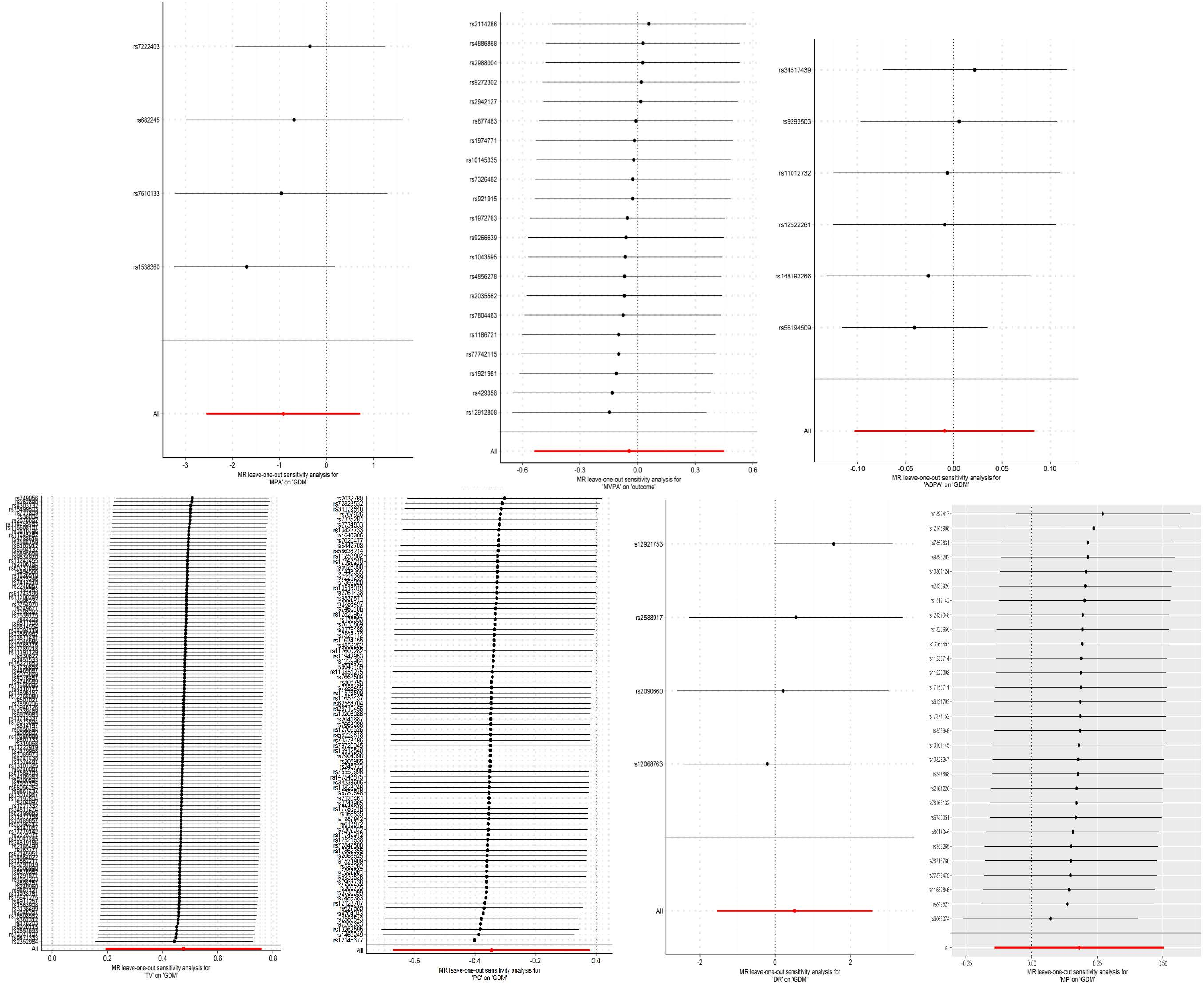

rs7222403
rs682245
rs7610133
rs1538360
All
-3
-2
-1
0
1
MR leave-one-out sensitivity analysis for
'MPA' on 'GDM'
rs2114286
rs4886868
rs2988004
rs9272302
rs2942127
rs877483
rs1974771
rs10145335
rs7326482
rs921915
rs1972763
rs9266639
rs1043595
rs4856278
rs2035562
rs7804463
rs1186721
rs77742115
rs1921981
rs429358
rs12912808
All
-0.6
-0.3
0.0
0.3
0.6
MR leave-one-out sensitivity analysis for
'MVPA' on 'outcome'
rs34517439
rs9293503
rs11012732
rs12522261
rs148193266
rs56194509
All
-0.10
-0.05
0.00
0.05
0.10
MR leave-one-out sensitivity analysis for
'ABPA' on 'GDM'
All
0.0
0.2
0.4
0.6
0.8
MR leave-one-out sensitivity analysis for
'TV' on 'GDM'
All
-0.6
-0.4
-0.2
0.0
MR leave-one-out sensitivity analysis for
'PC' on 'GDM'
rs12921753
rs2588917
rs2090660
rs12068763
All
-2
0
2
MR leave-one-out sensitivity analysis for
'DR' on 'GDM'
All
-0.25
0.00
0.25
0.50
MR leave-one-out sensitivity analysis for
'MP' on 'GDM'
