# Supplementary material for: Association between physical activity and sedentary behavior and gestational diabetes mellitus: a Mendelian randomization analysis
Source: Front Endocrinol (Lausanne). 2024 Dec 16;15:1389453. doi: 10.3389/fendo.2024.1389453 (PMC11682963; doi:10.3389/fendo.2024.1389453)
Supplement: Supplementary file 2 [file SupplementaryFile2.docx]

**STROBE-MR checklist of recommended items to address in reports of Mendelian randomization studies**^1^ ^2^

| **Item No.** | **Section** | **Checklist item** | **Page No.** | **Relevant text from manuscript** |
| --- | --- | --- | --- | --- |
| 1 | **TITLE and ABSTRACT** | Association between physical activity and sedentary behavior and gestational diabetes mellitus: A Mendelian randomization analysis | - | - |
|  | **INTRODUCTION** |  | - | - |
| 2 | **Background** | Gestational diabetes mellitus (GDM) is a common metabolic disease of pregnancy that increases the risk of adverse pregnancy and neonatal outcomes. Some studies have demonstrated that physical activity (PA) and sedentary behavior (SB) also influence the incidence of GDM by influencing BMI, but remains controversial in observational studies. MR analysis is superior to randomized controlled trials in several respects. First, Mendelian randomization (MR) analysis can remove the effects of confounding factors and reverse causality, enabling the examination of the cause-effect relationship between exposure and outcome. Secondly, MR can also save time and reduce economic costs while obtaining a larger sample size. To investigate the association between PA, SB, BMI and GDM, we designed univariate MR (UVMR), multivariable MR (MVMR) and Mediation MR studies. | - | - |
| 3 | **Objectives** | The objective of this study was threefold. First, we sought to investigate the potential relationship between PA, SB and GDM through UVMR studies. Second, we determined whether PA, SB was responsible for the onset and progression of GDM through MVMR, and finally, we determined whether BMI had a mediating effect between the two through Mediation MR. | - | - |
|  | **METHODS** |  | - | - |
| 4 | **Study design and data sources** | Present key elements of the study design early in the article. Consider including a table listing sources of data for all phases of the study. For each data source contributing to the analysis, describe the following: | - | - |
|  | a) | Setting: A univariate MR (UVMR) study was performed to determine the relationship between PA, SB and GDM. We also performed multivariate MR (MVMR) analysis to reduce the interference of confounding variables. On the results. This study is based on a European population. The research design process is illustrated in Figure 1. | - | - |
|  | b) | Participants: Specific information and sources of data for all populations are shown in Table 1. The participants summary statistical data of all exposures used in this study were retrieved from the largest research by Klimentidis et al. The GDM population was defined using Finnish health and population registry sources including registry data from inpatient hospitalizations, outpatient specialty clinics, and birth registry with diagnosed with ICD9, ICD10 within the gestational window (40 weeks before to 5 weeks after delivery) and excluding diabetes diagnosed before the first pregnancy, including 173,746 individuals (9,370 cases and 16,437 controls) of European ancestry. | - | - |
|  | c) | we determined the following selection criteria to reduce the likelihood of missing SNPs while maintaining a high correlation with exposure: P-value < 5×10-8, linkage disequilibrium (LD) r2 < 0.1, and window kb = 10,000. The SNPs used as IVs are listed in Supplementary Tables 1. F-statistics for every SNP were also produced to evaluate the usefulness of the IVs. More precisely, a powerful instrument is typically indicated by an F-statistic greater than 10, which implies that IVs are predictive of the exposure variable. | - | - |
|  | d) | PA is mainly defined by questionnaires: (1) For MPA, participants were asked: “In a typical WEEK, on how many days did you do 10 minutes or more of moderate physical activities like carrying light loads, cycling at normal pace? (Do not include walking)”. We assessed PA during the first trimester of pregnancy, as early pregnancy provides a critical baseline for evaluating lifestyle factors that may influence gestational outcomes. PA levels tend to decline as pregnancy progresses due to increasing physical discomfort and other factors. Evaluating PA in early pregnancy allows us to capture habits that may have a significant impact on the development of GDM. The implications of assessing PA at different stages of pregnancy are crucial: while PA in early pregnancy may reflect pre-pregnancy behavior, PA in the second and third trimesters is often more limited, which can diminish its potential protective effects against GDM. (2) For MVPA, this activity was calculated by taking the sum of total minutes/week of MPA multiplied by four and the total number of vigorous PA (which also defined by questionnaires) minutes/week multiplied by eight, corresponding to their metabolic equivalents. (3) For ABPA, participants were told to wear an Axivity AX3 wrist-worn accelerometer and began monitoring for up to 7 days, physical activity information was extracted from 100Hz raw triaxial acceleration data after calibration, removal of gravity, sensor noise, and identification of wear/non-wear episodes. Individuals with less than 72 hours of data or no data for each hour of a 24-hour period and outliers with values more than 4 standard deviations above the mean were excluded. SB was also determined by asking three questions: (1) For TV, participants were asked: “In a typical DAY, how many hours do you spend watching TV?”; (2) For PC, participants were asked: “In a typical DAY, how many hours do you spend using the computer? (Do not include using a computer at work)”. (3) For DR, participants were asked: “In a typical DAY, how many hours do you spend driving?”. The duration of these sedentary behaviors was treated as exposure measurements. (4) For MP, participants were asked: “In a typical DAY, how many hours do you spend using mobile phones?”. The duration of these sedentary behaviors was treated as exposure measurements.  The GDM population was defined using Finnish health and population registry sources including registry data from inpatient hospitalizations, outpatient specialty clinics, and birth registry with diagnosed with ICD9, ICD10 within the gestational window (40 weeks before to 5 weeks after delivery) and excluding diabetes diagnosed before the first pregnancy. | - | - |
|  | e) | All participating studies of GWAS have obtained approval from relevant institutional review boards, and written informed consent was received from all subjects. | - | - |
| 5 | **Assumptions** | To render the selected IVs for MR analysis comprehensive, three assumptions must be made: (1) The SNPs utilized as IVs should have a high correlation with exposure. Therefore, maintaining a clear logical structure when presenting these points is essential. (2) Genetic variation has no bearing on the possible genetic or environmental factors that could influence the outcome. (3) IVs should only affect the outcomes through exposure. | - | - |
| 6 | **Statistical methods: main analysis** | Describe statistical methods and statistics used |  |  |
|  | a) | There are no quantitative variables in the analyses. | - | - |
|  | b) | we determined the following selection criteria to reduce the likelihood of missing SNPs while maintaining a high correlation with exposure: P-value < 5×10-8, linkage disequilibrium (LD) r2 < 0.1, and window kb = 10,000. The SNPs used as IVs are listed in Supplementary Tables 1. F-statistics for every SNP were also produced to evaluate the usefulness of the IVs. More precisely, a powerful instrument is typically indicated by an F-statistic greater than 10, which implies that IVs are predictive of the exposure variable. | - | - |
|  | c) | we used the inverse variance weighted (IVW) method to assess the association between exposure and outcome, which provided the highest estimated power and chose the weighted median method for supplementary analysis. | - | - |
|  | d) | The data utilized in the study were derived from the comprehensive GWAS study and did not exhibit any instances of missing values. Furthermore, during the screening process for instrumental variables, missing values are excluded from the study because they do not meet the selection criteria for instrumental variables. | - | - |
|  | e) | If applicable, indicate how multiple testing was addressed | NA | NA |
| 7 | **Assessment of assumptions** | We set the selection criteria: (1)P-value < 5×10-8 (2) linkage disequilibrium (LD) r2 < 0.1(3) and window kb = 10,000 (4)F-statistic greater than 10. | - | - |
| 8 | **Sensitivity analyses and additional analyses** | Heterogeneity among the SNPs in IVW computation was assessed using Cochran's Q test. If the P-value was greater than 0.05, the assumption of existing heterogeneity among SNPs was rejected; otherwise, a random-effects model was used. Subsequently, the MR-Egger intercept test was performed to identify horizontal pleiotropic effects. If the P-value obtained by this test was < 0.05, the selected SNPs influenced multiple phenotypes. The MR-PRESSO test was used for global and distortion testing. Heterogeneity was considered present if the results were statistically significant. The total effect of each remaining SNP was estimated using the leave-one-out methodology. | - | - |
| 9 | **Software and pre-registration** |  |  |  |
|  | a) | Software: The R package “TwoSampleMR” (version 0.5.6) in the R software (version 4.1.3) was used for analysis. | - | - |
|  | b) | The study protocol and details have been pre-registered in Lanzhou University. | - | - |
|  | **RESULTS** |  |  |  |
| 10 | **Descriptive data** |  |  |  |
|  | a) | Report the numbers of individuals at each stage of included studies and reasons for exclusion. Consider use of a flow diagram | - | - |
|  | b) | Summary statistics for phenotypic exposure(s), outcome(s), and other relevant variables (e.g. means, SDs) are reported in Table 1 and supplementary table1. | - | - |
|  | c) | There are no meta-analyses of previous studies included in the data sources. | - | - |
|  | d) | For two-sample MR:  i.  We have set the selection criteria:P-value < 5×10-8, linkage disequilibrium (LD) r^2^ < 0.1, and window kb = 10,000.  ii.  To reduce the number of individuals who overlap between the exposure and outcome studies, we chose data from different databases for exposure and outcome, respectively. | - | - |
| 11 | **Main results** |  |  |  |
|  | a) | The p-value for each SNP is less than 0.05, and the F-statistic for each SNP is greater than 10. We screened SNPs closely related to exposure, including MPA4, MVPA22, ABPA6, TV123, PC85, MP31, and DR6, as instrumental variables. | - | - |
|  | b) | MR estimates of the relationship between exposure and outcome and odds ratios See Figures 2, 3, and 4 for details. | - | - |
|  | c) | If relevant, consider translating estimates of relative risk into absolute risk for a meaningful time period | NA | NA |
|  | d) | The graphical representation of the visualisation results is specified in Figures 2, 3, 4 and supplementary figures. | - | - |
| 12 | **Assessment of assumptions** |  |  |  |
|  | a) | The assessment of the validity of the hypotheses is illustrated in Figures 2, 3 and 4 and in the results section. | - | - |
|  | b) | The additional statistics (e.g., assessments of heterogeneity across genetic variants, such as *I^2^*, Q statistic or E-value) is seen in supplementary materials. | - | - |
| 13 | **Sensitivity analyses and additional analyses** |  |  |  |
|  | a) | Sensitivity analyses included Cochran's Q test and horizontal pleiotropy testing (Figure 2). Heterogeneity was present in all analyses, as indicated by a P-value < 0.05. The MR-PRESSO test results showed that there was no heterogeneity or outliers in any of the datasets. The MR effect magnitude was re-estimated using random-effects models, and causality was verified. The MR-Egger intercept tests had P > 0.05, which suggests that horizontal pleiotropy was not significant (Figure 2). Ultimately, the leave-one-out analysis and visualization supported the reliability of our findings (supplementary figures). | - | - |
|  | b) | The sensitivity analyses or additional analyses were seen in supplementary materials. | - | - |
|  | c) | In univariate MR analysis, we found that genetically predicted TV and PC among SB were associated with GDM (OR = 1.61, 95%CI 1.21-2.14, P = 0.001; OR = 0.71, 95%CI 0.51-0.98, P = 0.037), whereas DR and MP were not (OR = 1.68, 95%CI 0.21-13.3, P = 0.623; OR = 1.20, 95%CI 0.87-1.65, P = 0.271). However, no association was found between physical activity and GDM: MPA (OR = 0.40, 95%CI 0.08-2.06, P = 0.273), MVPA (OR = 0.96, 95%CI 0.58-1.57, P = 0.861), and ABPA (OR = 0.99, 95%CI 0.90-1.09, P = 0.838). Multivariate MR analysis found that TV is independently and positively associated with the risk of GDM (OR = 1.64, 95%CI 1.13-2.36, P = 0.008) and that BMI was a mediating factor with a 62% mediating effect. | - | - |
|  | d) | Our findings are consistent with those of previous studies. Our study had similar results as a recent MR study, which validated that TV can more than double the incidence of T2DM (OR: 2.3490, 95% CI 1.9084–2.8915, P value < 0.0001). This result was also validated by a large meta-analysis in which TV was positively associated with T2DM (OR 1.09, 95% CI 1.07–1.12), and this effect was independent of total sedentary time. In a prospective study of GDM, the authors investigated 188 pregnant women and found that the duration of TV was positively associated with GDM (OR 3.03, 95% CI 1.21–7.96). We found that BMI was an important mediator in the association between TV and GDM, with a mediating effect of 62% and that the association between TV and GDM was no longer significant after adjusting for BMI, which is similar to the results of previous studies. Zhang reported that television watching increased the risk of GDM (OR 1.74, 95% CI 1.29-2.34), but the relationship disappeared after correction for BMI. A prospective cohort study by Akilew et al. comparing obese women in the pre-pregnancy period to women with normal BMI found that baseline obesity was associated with a 76% (95% CI 1.11-2.80) increased risk of GDM. | - | - |
|  | e) | The additional plots to visualize results (e.g., leave-one-out analyses) were seen in supplementary figures. | - | - |
|  | **DISCUSSION** |  |  |  |
| 14 | **Key results** | This study proposes a new hypothesis for the association between TV and GDM, which is mediated by BMI, providing evidence for reducing the risk of GDM during pregnancy by reducing television watching time. | - | - |
| 15 | **Limitations** | Firstly, we have not found any association between PA and GDM, but previous studies have demonstrated that mild PA attenuates endothelial impairment in the pathogenesis of diabetes and reduces cardiovascular events or all-cause mortality, which we did not analyse further because of the unavailability of these data in the current GWAS database, which requires future studies to confirm. Secondly, because the ABPA is measured subjectively, there may be some statistical data error. Moreover, we found a negative correlation between PC and GDM in UVMR, although it was excluded from the follow-up analysis, which is worthy of consideration and does not exclude the possibility that pregnant individuals who use computers have a higher cognitive ability and some knowledge of preventive measures for GDM; this requires further research on the relationship between pregnancy demographic characteristics (e.g., cognition, education, occupation, etc.) and the incidence of GDM [63]. There are also other forms of SB, such as reading, which we did not include in the analysis because there are no GWAS data captured from the same sequencing platform and the same cohort of the population and will also require additional research in the future. In addition, the population data we included for the GDM was pregnant, and characteristics such as age were not provided in the original data, the applicability of the conclusions of this study to the pre-pregnancy population or to different age groups may require more careful judgment. Finally, owing to the strict screening criteria, we obtained fewer instrumental variables for partial exposure in UVMR, which should be addressed in future with updated summary data. | - | - |
| 16 | **Interpretation** |  | - | - |
|  | a) | Meaning: our study provides evidence of an association between television watching and GDM, with BMI acting as a significant mediator. However, our findings for other types of SB, such as computer use and driving, did not reach statistical significance, and PA did not show a clear association with GDM. Given these results, further studies are necessary to verify the role of different types of SB and PA in GDM risk, particularly using more objective measures and larger datasets.  This study contributes to the growing body of evidence linking SB with GDM but highlights the need for additional research to confirm the associations for other SB types and PA, as well as to explore the potential combined effects of PA and SB on GDM. Future research should also consider the timing of PA and SB assessments to fully understand their role throughout pregnancy. These findings support ongoing public health efforts to reduce sedentary behaviour and promote regular physical activity during pregnancy to mitigate the risk of GDM. | - | - |
|  | b) | Mechanism: This association may be related to the following mechanisms: (1) TV watching represents low energy expenditure (resting metabolic rate or ≤1.5 metabolic equivalents) and predisposes to eating habits that produce unhealthy extra energy intake, which results in energy imbalance (energy intake > energy expenditure) and leads to the development of GDM. (2) In addition, the tendency to consume ultra processed foods, which are high in carbohydrates and fat, while watching television can adversely affect the control of 2-h postprandial glucose, fasting glucose, and lipids, even if the total daily energy intake is not exceeded. (3) BMI measured early in pregnancy was shown to mediate a significant portion of the risk associated with sedentary behaviours and the development of GDM. This aligns with prior research indicating that early pregnancy BMI, combined with weight gain during the second and third trimesters, plays a pivotal role in GDM risk. These factors jointly promote the development of obesity while watching television and contribute to the development of GDM. | - | - |
|  | c) | Clinical relevance: In terms of clinical management, TV as a specific sedentary behaviour is associated with the largest effect value for GDM compared to PA and other types of SB, and the importance of controlling SB in addition to increasing PA for the prevention of GDM has been emphasized in the public health guidelines, especially as some people with activity difficulties during pregnancy will inevitably reduce PA, and our results provide supporting evidence to guide clinical practice for the population. Through changes in effect sizes in the mediation analyses, we found that it is possible to reduce BMI, and thus the risk of GDM, by reducing television watching time in pregnant women, whereas simply reducing the television watching time without controlling or even increasing BMI (e.g., increasing intake) does not appear to prevent GDM. It emphasizes the importance of the risk of GDM associated with overweight or obesity and offers evidence for clinical healthcare providers to guide the practice of pregnant and pregnancy preparation populations with new recommendations that simply reducing prolonged sedentary time does not relax the management of BMI, which provides important information to guide clinical interventions for the prevention and treatment of GDM. | - | - |
| 17 | **Generalizability** | This study is based on a European population and we will include other populations in future studies to increase the generalizability of the results. | - | - |
|  | **OTHER INFORMATION** |  | - | - |
| 18 | **Funding** | This study was funded by National Natural Science Foundation of China (No.81960155). | - | - |
| 19 | **Data and data sharing** | The GWAS data used in this work are publicly available at the IEU Open GWAS Project (https://gwas.mrcieu.ac.uk/. The accession code as follows: MPA, ukb-b-2346; MVPA, ebi-a-GCST006097; ABPA, ebi-a-GCST006099; TV, ukb-b-5192; PC, ukb-b-4522; DR, ukb-b-3793; MP, ukb-b-4094; BMI, ukb-b-19953) and the FinnGen GWAS results (https://r7.finngen.fi/. The accession code is GEST_DIABETES). | - | - |
| 20 | **Conflicts of Interest** | All authors declare that there were no potential conflicts of interest. | - | - |

This checklist is copyrighted by the Equator Network under the Creative Commons Attribution 3.0 Unported (CC BY 3.0) license.

1. Skrivankova VW, Richmond RC, Woolf BAR, Yarmolinsky J, Davies NM, Swanson SA, et al. Strengthening the Reporting of Observational Studies in Epidemiology using Mendelian Randomization (STROBE-MR) Statement. JAMA. 2021;under review.

2. Skrivankova VW, Richmond RC, Woolf BAR, Davies NM, Swanson SA, VanderWeele TJ, et al. Strengthening the Reporting of Observational Studies in Epidemiology using Mendelian Randomisation (STROBE-MR): Explanation and Elaboration. BMJ. 2021;375:n2233.
